# Supplementary material for: The neurodevelopmental trajectory of beta band oscillations: an OPM-MEG study
Source: bioRxiv. 2024 Mar 19:2024.01.04.573933. Originally published 2024 Jan 4. Preprint. [Version 2] doi: 10.1101/2024.01.04.573933 (PMC10802362; doi:10.1101/2024.01.04.573933)
Supplement: Supplement 1 [file NIHPP2024.01.04.573933v2-supplement-1.pdf]

bioRxiv preprint doi: https://doi.org/10.1101/2024.01.04.573933; this version posted March 19, 2024. The copyright holder for this preprint (which was not certified by peer review) is the author/funder, who has granted bioRxiv a license to display the preprint in perpetuity. It is made available under aCC-BY 4.0 International license.

The neurodevelopmental trajectory of beta band oscillations: an OPM-MEG study

Supplementary Information

Lukas Rier, Natalie Rhodes, Daisie Pakenham, Elena Boto, Niall Holmes, Ryan M. Hill, Gonzalo Reina Rivero, Vishal Shah, Cody Doyle, James Osborne, Richard Bowtell, Margot J. Taylor and Matthew J. Brookes

Section 1: Beta modulation with stimulation of the little finger:

Figure 3 shows beta modulation as a function of age, following tactile stimulation of the index finger. The same methods were used to generate equivalent responses during trials in which the little finger received stimulation. This is shown in Figure S1, which supports the conclusions drawn in the main manuscript. Again, a significant variation of beta modulation with age is observed.

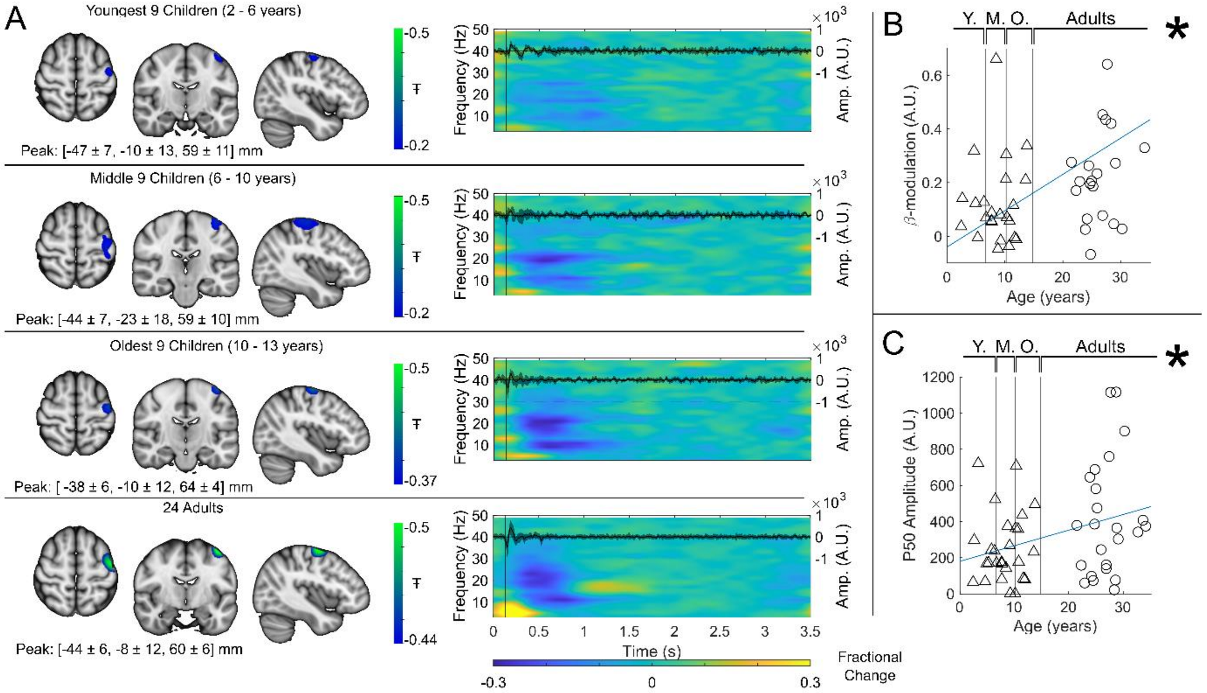

Figure S1: Equivalent to Figure 3 in the main manuscript but derived for little finger (rather than index finger) stimulation. Beta modulation showed a significant increase with age ($R^2 = 0.23, p = 0.0003$).The P50 Amplitude showed a weak but significant increase with age ($R^2 = 0.1, p = 0.023$).

bioRxiv preprint doi: https://doi.org/10.1101/2024.01.04.573933; this version posted March 19, 2024. The copyright holder for this preprint (which was not certified by peer review) is the author/funder, who has granted bioRxiv a license to display the preprint in perpetuity. It is made available under aCC-BY 4.0 International license.

Section 2: burst amplitude does not correlate with age.

Our manuscript shows a significant correlation between beta modulation and burst probability (Figure 5D) – implying that the stimulus related drop in beta amplitude occurs because bursts are less likely to occur during this window. Further, we showed significant age-related changes in both beta amplitude modulation and burst probability, leading to a conclusion that the age dependence of beta modulation was caused by changes in the likelihood of bursts (i.e. bursts are less likely to 'switch off' during sensory stimulation, in children). Here we extend these analyses to test whether burst amplitude also changes significantly with age. We reasoned that if burst amplitude remained the same in children and adults, this would not only suggest that beta modulation is driven solely by burst probability (distinct from children having lower amplitude bursts), but also show directly that the beta effects we see are not attributable to a lack of sensitivity in younger people.

We took the (unnormalized) beamformer projected electrophysiological time series from sensorimotor cortex and filtered them 5-48 Hz. (The motivation for the large band was because bursts are known to be pan-spectral and have lower frequency content in Children – this band captures most of the range of burst frequencies highlighted in our spectra.) We then extracted the timings of the bursts, and for each burst took the maximum projected signal amplitude. These values were averaged across all bursts in an individual subject and plotted for all subjects against age.

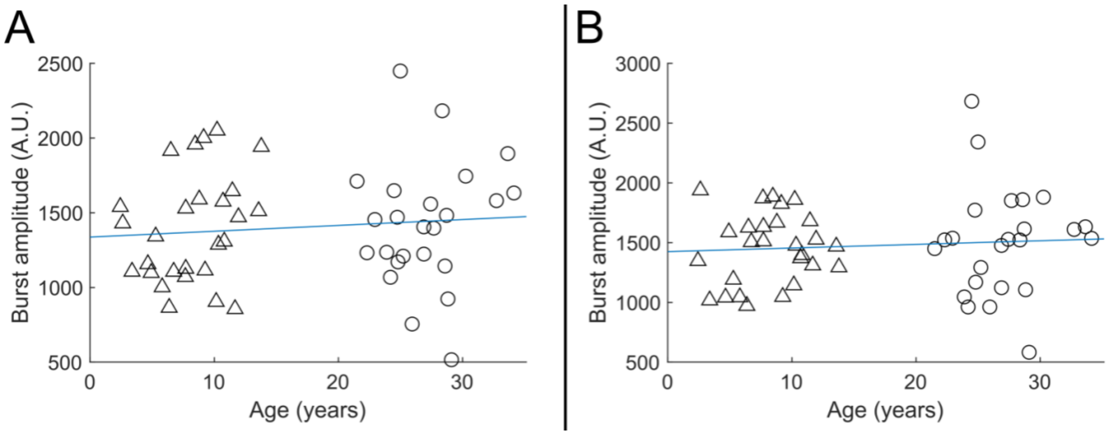

Figure S2: Beta burst amplitude as a function of age; A) shows index finger simulation trials (R^2 = 0.01, p = 0.48)*; B shows little finger stimulation trials (*R^2 = 0.01, p = 0.57)*. In both cases there was no significant modulation of burst amplitude with age.*

Results (see Figure S2) showed that the amplitude of the beta bursts showed no significant age-related modulation (R^2 = 0.01, p = 0.48 for the index finger (Panel A) and R^2 = 0.01, p = 0.57 for the little finger (Panel B)). This is distinct from both burst probability and task-induced beta modulation. This adds weight to the argument that the diminished beta modulation in children is not caused by a lack of sensitivity to the MEG signal and supports the conclusion that burst probability is the primary driver of age-related changes in beta oscillations.

bioRxiv preprint doi: https://doi.org/10.1101/2024.01.04.573933; this version posted March 19, 2024. The copyright holder for this preprint (which was not certified by peer review) is the author/funder, who has granted bioRxiv a license to display the preprint in perpetuity. It is made available under aCC-BY 4.0 International license.

Section 3: Burst spectra in the non-burst states.

Figure 6 in the main manuscript shows the frequency spectrum of the HMM-derived burst state. Here we show equivalent spectra for the non-burst states. As shown, even in the non-burst states, a change in spectrum is observed with children exhibiting less high-frequency and more low-frequency activity.

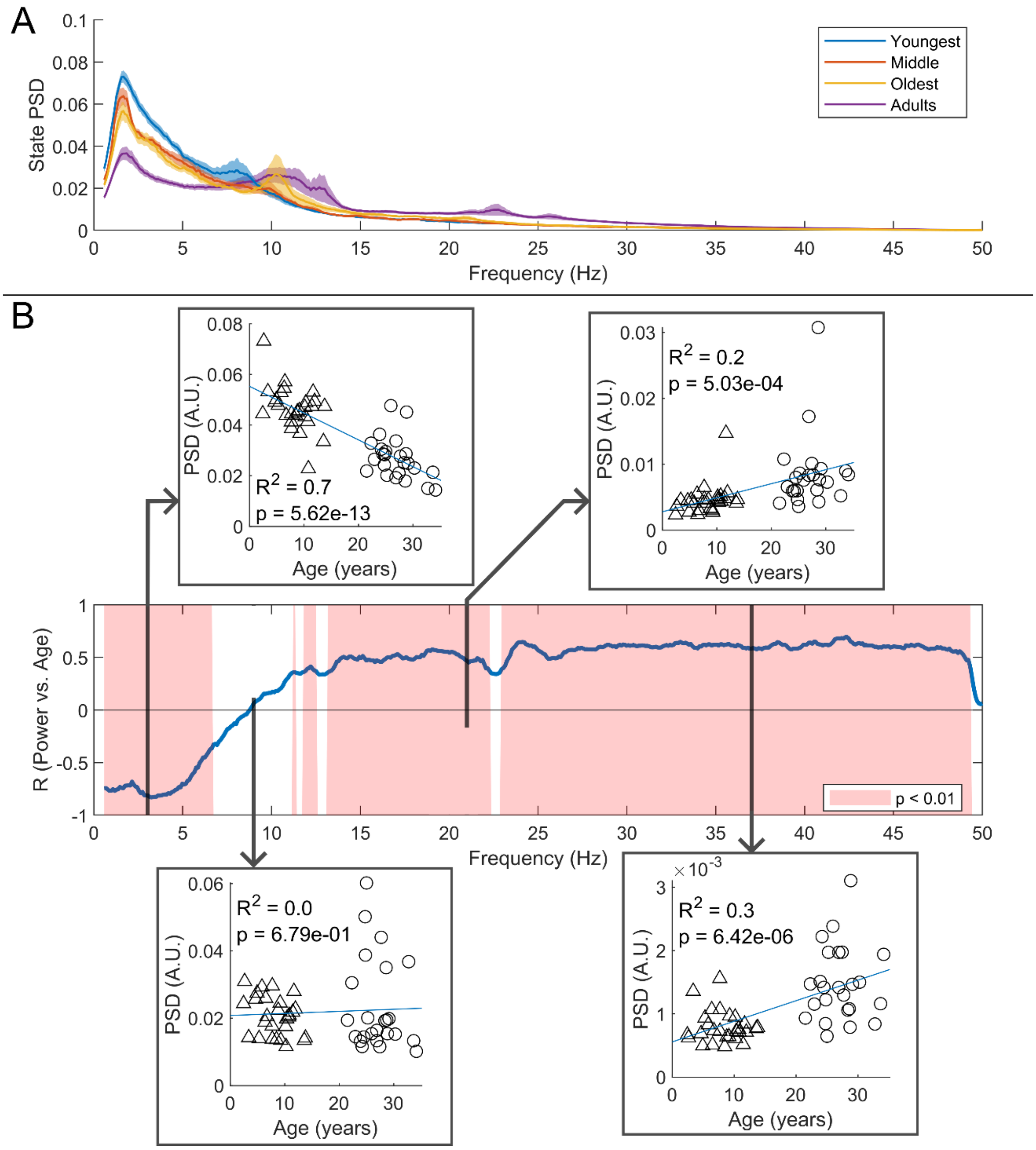

Figure S3: Spectral content of the non-burst states. (A) Average ***non-****burst-state spectra across groups. Shaded areas indicate standard error on the group mean. (B) Pearson correlation coefficient for the PSD values in (A) against age across all frequency values. Red shaded areas indicate $p < 0.01$ (uncorrected). The four inset plots show example scatters of PSD values with age at select frequencies (3 Hz, 9 Hz, 21 Hz, and 37 Hz). Low-frequency spectral content decreases with age while high-frequency content increases. Results broadly mirror the frequency content and age relationships found in the burst state, however, features in the spectra corresponding to classical alpha and beta peaks are less prominent outside the burst state.*

bioRxiv preprint doi: https://doi.org/10.1101/2024.01.04.573933; this version posted March 19, 2024. The copyright holder for this preprint (which was not certified by peer review) is the author/funder, who has granted bioRxiv a license to display the preprint in perpetuity. It is made available under aCC-BY 4.0 International license.

Section 4: Proximity of sensors to the head:

For an ideal wearable MEG system, the distance between the sensors and the scalp surface (sensor proximity) would be the same regardless of age (and head shape/size), ensuring maximum sensitivity in all subjects. To test how our system performed in this regard, we undertook analyses to compute scalp-to-sensor distances. This was done in two ways:

1. **Real distances in our adaptable system:** We took the co-registered OPM sensor locations and computed the Euclidean distance from the centre of the sensitive volume (i.e. the centre of the vapour cell) to the closest point on the scalp surface. This was measured independently for all sensors, and an average across sensors was calculated. We repeated this for all participants (recall participants wore helmets of varying size and this adaptability should help minimise any relationship between sensor proximity and age).
2. **Simulated distances for a non-adaptable system:** Here, the aim was to see how proximity might have changed with age, had only a single helmet size been used. We first identified the single example subject with the largest head (scanned wearing the largest helmet) and extracted the scalp-to-sensor distances as above. For all other subjects, we used a rigid body transform to co-register their brain to that of the example subject (placing their head (virtually) inside the largest helmet). Proximity was then calculated as above and an average across sensors calculated. This was repeated for all participants.

In both analyses, sensor proximity was plotted against age and significant relationships probed using Pearson correlation.

In addition, we also wanted to probe the relationship between sensor proximity and head circumference. Head circumference was estimated as follows: the whole head MRI was binarised (to delineate the surface of the head); the axial slice with the largest area was selected and circumference of the head within that slice measured. We then plotted sensor proximity versus head circumference, for both the real (adaptive) and simulated (non-adaptive) case (expecting a negative relationship – i.e. larger heads mean closer sensor proximity). The slope of the relationship was measured and we used a permutation test to determine whether the use of adaptable helmets significantly lowered the identified slope (i.e. do adaptable helmets significantly improve sensor proximity in those with smaller head circumference).

Results are shown in Figure S4. We found no measurable relationship between sensor proximity and age ($r = -0.19$; $p = 0.17$) in the case of the real helmets (panel A). When simulating a non-adaptable helmet, we did see a significant effect of age on scalp-to-sensor distance ($r = -0.46$; $p = 0.001$; panel B). This demonstrates the advantage of the adaptability of OPM-MEG; without the ability to flexibly locate sensors, we would have a significant confound of sensor proximity.

Plotting sensor proximity against head circumference we found a significant negative relationship in both cases ($R = -0.37$; $p = 0.007$ and $R = -0.78$; $p = 0.000001$); however, the difference between slopes was significant according to a two-tailed permutation test ($p < 0.025$) suggesting that adaptable helmets do indeed improve sensor proximity, in those with smaller head circumference. This again shows the benefits of adaptability to head size.

bioRxiv preprint doi: https://doi.org/10.1101/2024.01.04.573933; this version posted March 19, 2024. The copyright holder for this preprint (which was not certified by peer review) is the author/funder, who has granted bioRxiv a license to display the preprint in perpetuity. It is made available under aCC-BY 4.0 International license.

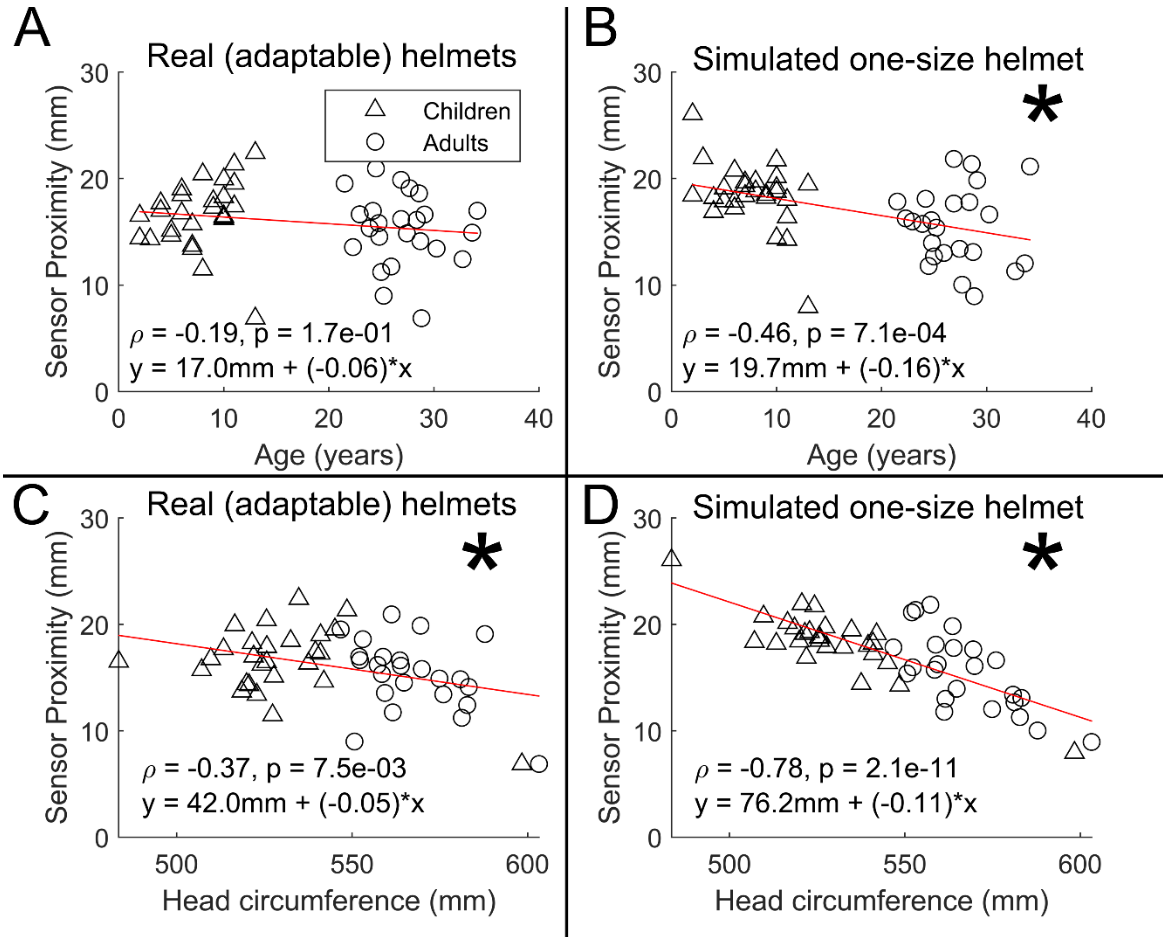

Figure S4: Scalp-to-sensor distance as a function of age (A/B) and head circumference (C/D). A and C show the case for the real helmets; B and D show the simulated non-adaptable case.

In sum, the ideal wearable system would see sensors located on the scalp surface, to get as close as possible to the brain in all subjects. Our system of multiple helmet sizes is not perfect in this regard (there is still a significant relationship between proximity and head circumference). However, our solution has offered a significant improvement over a (simulated) non-adaptable system. Future systems should aim to improve even further on this, either by using additively manufactured bespoke helmets for every subject (this is a gold standard, but also potentially costly for large studies), or adaptable flexible helmets.

bioRxiv preprint doi: https://doi.org/10.1101/2024.01.04.573933; this version posted March 19, 2024. The copyright holder for this preprint (which was not certified by peer review) is the author/funder, who has granted bioRxiv a license to display the preprint in perpetuity. It is made available under aCC-BY 4.0 International license.

Section 5: Reduced trial analyses:

In our study we had to discard more trials in children than adults. This potentially means a confound with a larger signal to noise ratio in adults than children, which could affect the results. For this reason we reanalysed our data, discarding trials from the adults to ensure equal numbers (on average) in our adult and child cohorts. Results are shown in Figure S5. Panel A shows beta modulation with age (equivalent to Figure 3B); Panel B shows evoked response (M50) modulation with age (equivalent to Figure 3C); Panel C shows functional connectivity with age (equivalent to Figure 4B) and Panel D shows burst probability modulation with age (equivalent to Figure 5C). In all cases, the significant modulations with age captured in the main manuscript remain.

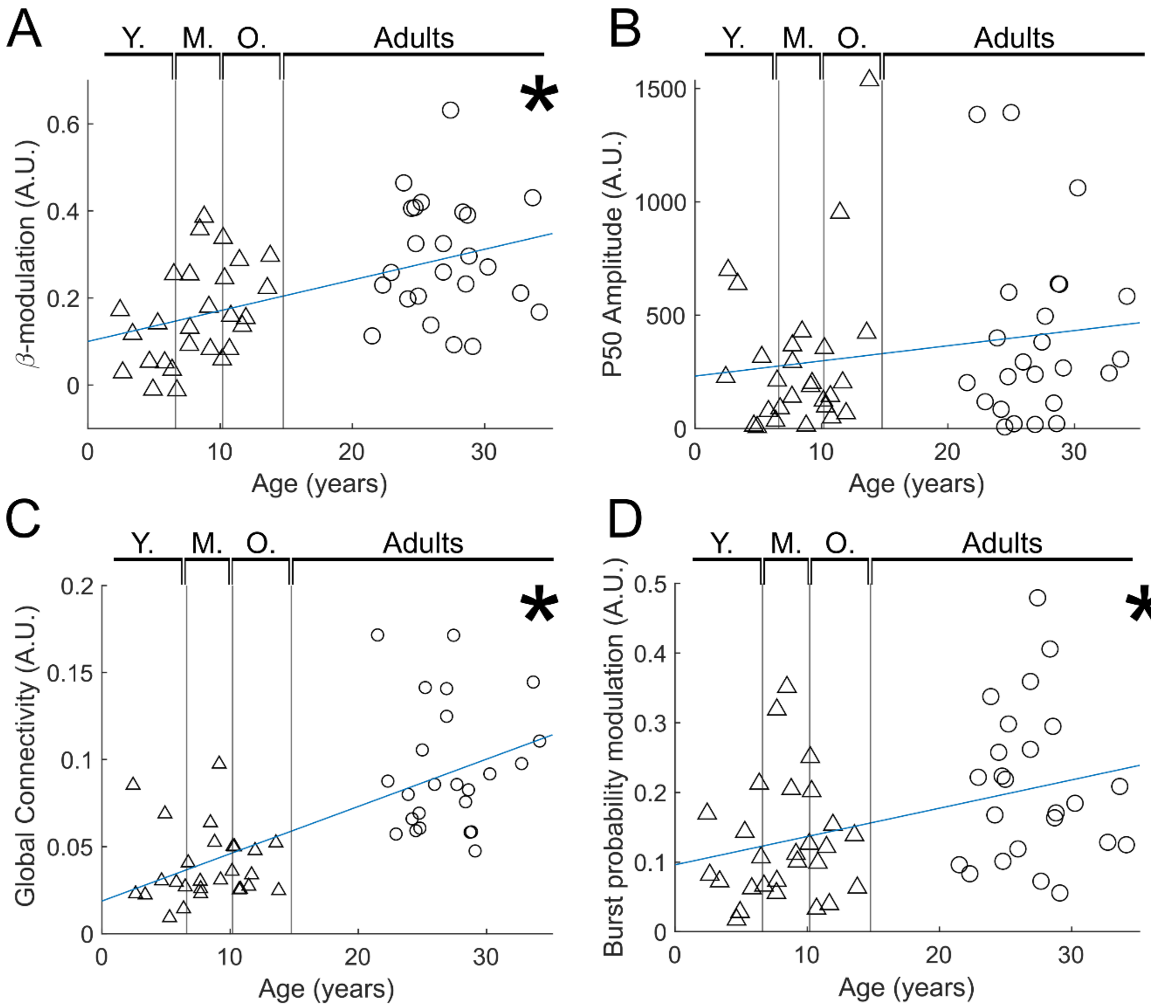

Figure S5: Reduced trial analysis for index finger stimuli: A) Beta modulation with age. ($R^2 = 0.26, p = 0.00014$) B) Evoked response (P50) modulation with age. ($R^2 = 0.03, p = 0.199$) C) Functional connectivity with age. ($R^2 = 0.45, p = 7 \times 10^{-8}$) D) Burst probability modulation with age ($R^2 = 0.15, p = 5.4 \times 10^{-3}$)
