# Supplementary material for: The neurodevelopmental trajectory of beta band oscillations: an OPM-MEG study
Source: bioRxiv. 2024 Jan 4:2024.01.04.573933. Preprint. [Version 1] doi: 10.1101/2024.01.04.573933 (PMC10802362; doi:10.1101/2024.01.04.573933)
Supplement: Supplementary file 1 [file NIHPP2024.01.04.573933v1-supplement-1.pdf]

bioRxiv preprint doi: https://doi.org/10.1101/2024.01.04.573933; this version posted January 4, 2024. The copyright holder for this preprint (which was not certified by peer review) is the author/funder, who has granted bioRxiv a license to display the preprint in perpetuity. It is made available under aCC-BY 4.0 International license.

THE NEURODEVELOPMENTAL TRAJECTORY OF BETA BAND OSCILLATIONS: AN OPM-MEG STUDY

SUPPLEMENTARY INFORMATION

Lukas Rier+, Natalie Rhodes+, Daisie Pakenham, Elena Boto, Niall Holmes, Ryan M. Hill, Gonzalo Reina Rivero, Vishal Shah, Cody Doyle, James Osborne, Richard Bowtell, Margot J. Taylor and Matthew J. Brookes

+Indicates authors who made equal contributions to this manuscript.

bioRxiv preprint doi: https://doi.org/10.1101/2024.01.04.573933; this version posted January 4, 2024. The copyright holder for this preprint (which was not certified by peer review) is the author/funder, who has granted bioRxiv a license to display the preprint in perpetuity. It is made available under aCC-BY 4.0 International license.

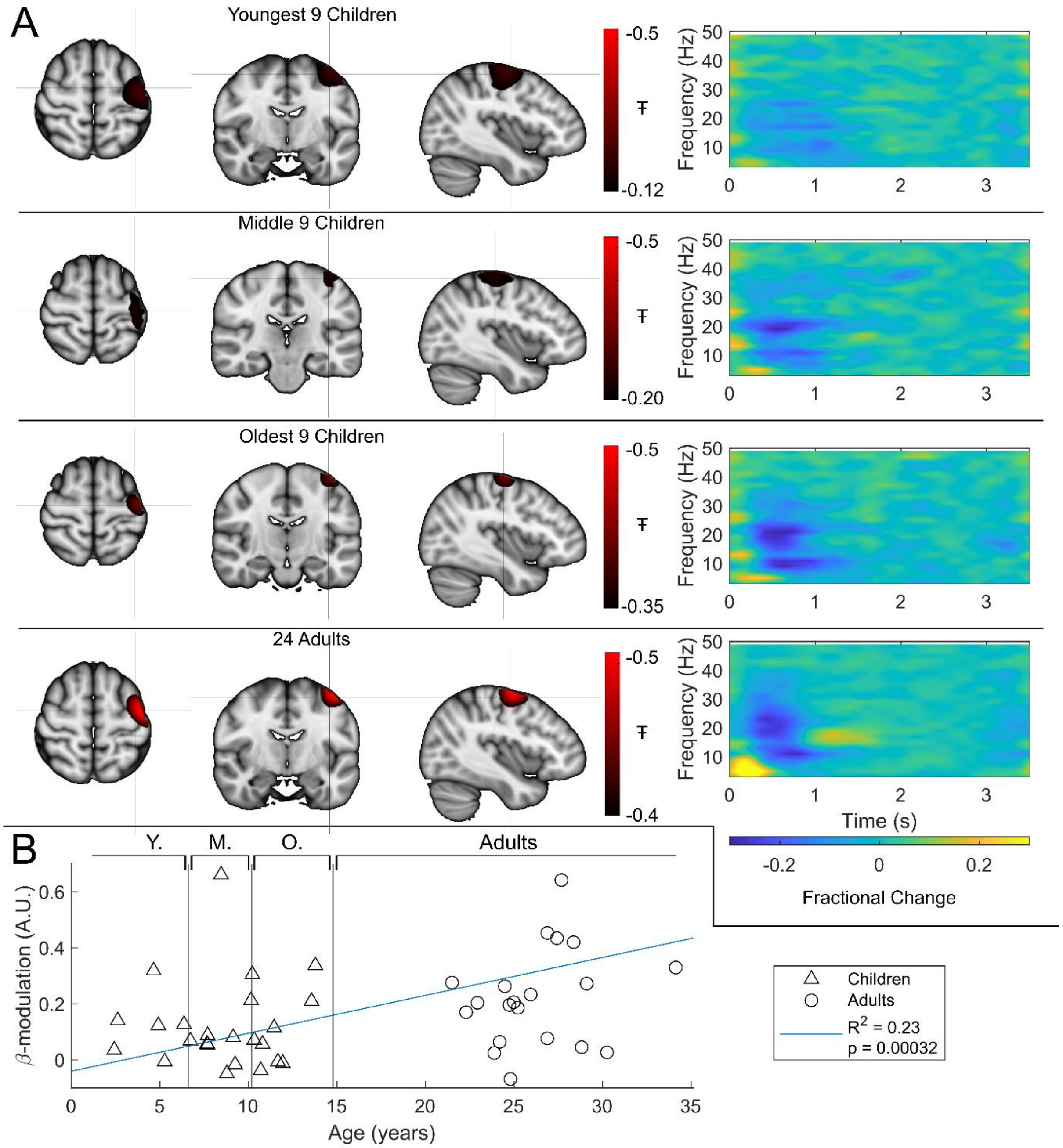

Figure S 1: Beta band modulation with age for little finger stimulation: *(A) Brain plots show slices through the left motor cortex, with a pseudo-T-statistical map of beta modulation (blue/black) overlaid on the standard brain. Time frequency spectrograms show modulation of the amplitude of neural oscillations (fractional change in spectral power relative to the baseline measured in the 2.5-3 s window). In all cases results were extracted from the location of peak beta power reduction during stimulation (in the left sensorimotor cortex). (B) Difference in beta-band amplitude (0.3-0.8 s window vs 1-1.5 s window) plotted as a function of age (i.e., each data point shows a different participant; triangles represent children, circles represent adults). Note the significant correlation* (R^2=0.23, p=0.00032*).

bioRxiv preprint doi: https://doi.org/10.1101/2024.01.04.573933; this version posted January 4, 2024. The copyright holder for this preprint (which was not certified by peer review) is the author/funder, who has granted bioRxiv a license to display the preprint in perpetuity. It is made available under aCC-BY 4.0 International license.

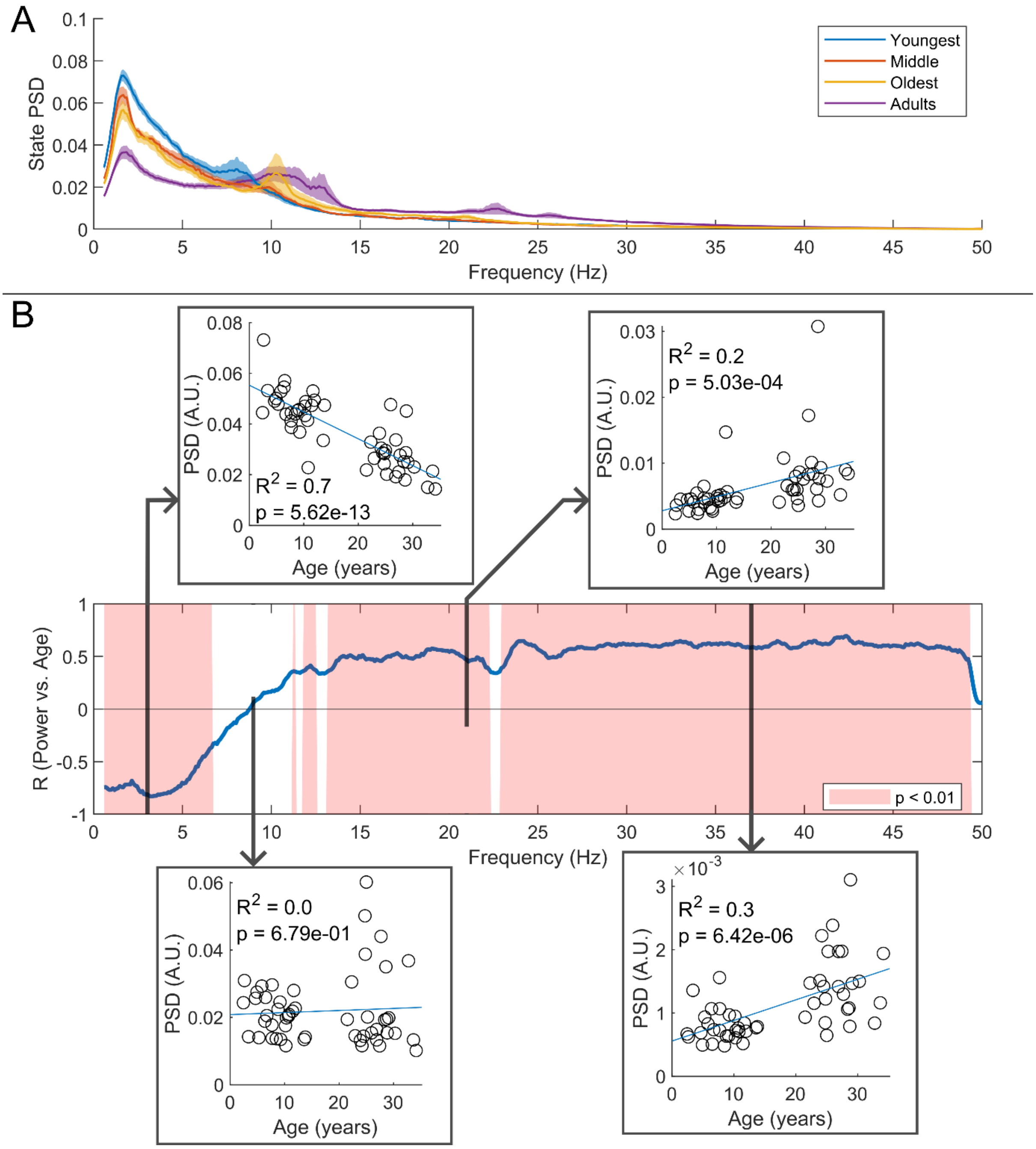

Figure S 2 Spectral content of the non-burst states. *(A) Average* ***non-****burst-state spectra across groups. Shaded areas indicate standard error on the group mean. (B) Pearson correlation coefficient for the PSD values in (A) against age across all frequency values. Red shaded areas indicate* $p < 0.01$ *(uncorrected). The four inset plots show example scatters of PSD values with age at select frequencies (3 Hz, 9 Hz, 21 Hz, and 37 Hz). Low-frequency spectral content decreases with age while high-frequency content increases. Results broadly mirror the frequency content and age relationships found in the burst state, however, features in the spectra corresponding to classical alpha and beta peaks are less prominent outside the burst state.*
